# Editorial Statement: *Forensic parasitology: a new frontier in criminalistics*

**DOI:** 10.1093/fsr/owae052

**Published:** 2025-09-12

This is an Editorial Statement regarding: *Forensic Sciences Research*, Volume 9, Issue 2, June 2024, owae005, https://doi.org/10.1093/fsr/owae005

In January 2024, *Forensic Sciences Research* (FSR) published the accepted manuscript of the article “*Forensic parasitology: a new frontier in criminalistics*” by Mackenzie L. Kwak, James F. Wallman, Darren Yeo, Melanie S. Archer, and Ryo Nakao.

https://doi.org/10.1093/fsr/owae005

In the final stage of editing before the publication of the corrected and typeset article in June 2024, the FSR Editorial Office removed the following italicized sentences from the uncorrected manuscript, without the authors’ approval, due to a miscommunication with the first author and the Editors-in-Chief:

Biological attacks using weaponized parasites represent a serious security threat which can lead to morbidity and mortalities in humans and other animals [30]. Such attacks can be perpetrated by states (e.g. security, intelligence, or defence ministries) or non-state actors (e.g. terrorist groups). Historically, numerous state and non-state actors have weaponized parasites (Table 2). *The United Nations’ Biological Weapons Convention was established in 1972 to reduce the proliferation of biological weapons by governments; however, in recent years a number of nations, including the People’s Republic of China, Russian Federation, Democratic People’s Republic of Korea and Islamic Republic of Iran, have been suspected of not complying with this UN convention [31]. Of these, there is strong evidence to suggest that the Democratic People’s Republic of Korea maintains an offensive biological weapons programme [31].* Therefore, a real possibility exists of parasites being weaponized and used by state actors. A wide range of terrorist organizations have also weaponized biological agents [32], as well as individual actors for criminal purposes [33].

*Reference 31: US Department of State. Adherence to and compliance with arms control, nonproliferation, and disarmament agreements and commitments. Compliance Report.*

*Reference 14: van Uhm D. Chinese wildlife trafficking networks along the silk road. In: Lo TW, Siege lD, Kwok SI (eds.), Organized Crime and Corruption across Borders: Exploring the Belt and Road Initiative. Abingdon (UK): Routledge, 2019, 114–133.*

The sentences in italics were removed because the Editorial Office staff thought the content could be interpreted as conflicting with the objectivity, neutrality, and impartiality that characterize the editorial principles of this scientific journal. We now publish this Editorial Statement, with our apologies to the authors for the miscommunication.

FSR Editorial Office

